# Optimizing the initial tacrolimus dosage in Chinese children with lung transplantation within normal hematocrit levels

**DOI:** 10.3389/fped.2024.1090455

**Published:** 2024-01-31

**Authors:** Ke Hu, Su-Mei He, Cun Zhang, Yi-Jia Zhang, Qian Gu, Hao-Zhe Shi, Dong-Dong Wang

**Affiliations:** ^1^ Jiangsu Key Laboratory of New Drug Research and Clinical Pharmacy & School of Pharmacy, Xuzhou Medical University, Xuzhou, Jiangsu, China; ^2^ Department of Pharmacy, Suzhou Hospital, Affiliated Hospital of Medical School, Nanjing University, Suzhou, Jiangsu, China; ^3^ Department of Pharmacy, Xuzhou Oriental Hospital Affiliated to Xuzhou Medical University, Xuzhou, Jiangsu, China

**Keywords:** optimizing, initial tacrolimus dosage, children, lung transplantation, normal hematocrit

## Abstract

**Background:**

The appropriate initial dosage of tacrolimus is undefined in Chinese pediatric lung transplant patients with normal hematocrit values. The purpose of this study is to optimize the initial dose of tacrolimus in Chinese children who are undergoing lung transplantation and have normal hematocrit levels.

**Methods:**

The present study is based on a published population pharmacokinetic model of tacrolimus in lung transplant patients and uses the Monte Carlo simulation to optimize the initial tacrolimus dosage in Chinese children with lung transplantation within normal hematocrit levels.

**Results:**

Within normal hematocrit levels, for children with lung transplantation who do not carry the CYP3A5*1 gene and have no coadministration with voriconazole, it is recommended to administer tacrolimus at a dosage of 0.02 mg/kg/day, divided into two doses, for children weighing 10–32 kg, and a dosage of 0.03 mg/kg/day, also divided into two doses, for children weighing 32–40 kg. For children with lung transplantation who carry the CYP3A5*1 gene and have no coadministration with voriconazole, tacrolimus dosages of 0.02, 0.03, and 0.04 mg/kg/day split into two doses are recommended for children weighing 10–15, 15–32, and 32–40 kg, respectively. For children with lung transplantation who do not carry the CYP3A5*1 gene and have coadministration with voriconazole, tacrolimus dosages of 0.01 and 0.02 mg/kg/day split into two doses are recommended for children weighing 10–17 and 17–40 kg, respectively. For children with lung transplantation who carry the CYP3A5*1 gene and have coadministration with voriconazole, a tacrolimus dosage of 0.02 mg/kg/day split into two doses is recommended for children weighing 10–40 kg.

**Conclusions:**

It is the first time to optimize the initial dosage of tacrolimus in Chinese children undergoing lung transplantation within normal hematocrit.

## Highlights

• For children with lung transplantation who do not carry the CYP3A5*1 gene and have no coadministration with voriconazole, the recommended dosages of tacrolimus are 0.02 and 0.03 mg/kg/day split into two doses for children weighing 10–32 and 32–40 kg, respectively.
• For children with lung transplantation who carry the CYP3A5*1 gene and have no coadministration with voriconazole, the recommended dosages of tacrolimus, are 0.02, 0.03, and 0.04 mg/kg/day split into two doses for children weighing 10–15, 15–32, and 32–40 kg, respectively.
• For children with lung transplantation who do not carry the CYP3A5*1 gene and have coadministration with voriconazole, the recommended dosages of tacrolimus are 0.01 and 0.02 mg/kg/day split into two doses for children weighing 10–17, 17–40 kg, respectively.
• For children with lung transplantation who carry the CYP3A5*1 gene and have coadministration with voriconazole, the recommended dosage of tacrolimus is 0.02 mg/kg/day split into two doses for children weighing 10–40 kg.
• It is the first time to optimize the initial tacrolimus dosage in children with lung transplantation within normal hematocrit.

## 1 Introduction

Lung transplantation is the only effective treatment for children with end-stage lung disease (1–3); however, for lung transplant recipients, the lungs after transplantation are often rejected by the recipient. If the rejection reaction after lung transplantation is not well controlled, it will bring serious threats to the lives of children receiving lung transplantation. Fortunately, tacrolimus-based immunosuppressant therapy can better control organ rejection after transplantation (4–7). Meanwhile, tacrolimus therapy has been widely used in hematopoietic stem cell transplantation (8), liver transplantation (7), kidney transplantation (6), heart transplantation (4), and lung transplantation (5).

The clinical efficacy and adverse reactions of tacrolimus are closely related to its drug concentrations, where higher tacrolimus concentrations increase the risk of toxicity and lower tacrolimus concentrations make efficacy less desirable (9, 10). However, tacrolimus is difficult to realize individualized drug administration clinically due to the large inter-individual and intra-individual pharmacokinetic variation and narrow therapeutic window (11–14), where many factors can influence the pharmacokinetic course of tacrolimus. For example, Zhao et al. (15) reported that tacrolimus exposure increased significantly during voriconazole co-therapy, and Chen et al. (8) similarly found that voriconazole significantly increased tacrolimus concentrations. In addition, Reininger et al. (16) reported higher number of tacrolimus dose adjustments in kidney transplant recipients who were extensive and intermediate *CYP3A5* metabolizers, and Chen et al. (17) recommended an optimal initial regimen of tacrolimus for children with systemic lupus erythematosus based on the *CYP3A5* polymorphism. In addition, Piletta-Zanin et al. (18) reported that low hematocrit levels can result in tacrolimus toxicity, and Schijvens et al. (19) reported the potential impact of hematocrit correction on the evaluation of tacrolimus target exposure in pediatric kidney transplant patients.

At present, some studies have been conducted on the individualized administration of tacrolimus in pediatric organ transplantation (20, 21). According to the International Society for Heart and Lung Transplantation, pulmonary vascular disease, including idiopathic pulmonary hypertension, is the most common indication of lung transplant recipients in children aged 1–5 years (22). Pulmonary hypertension is also one of the most common primary diseases in all children receiving lung transplantation in China, accounting for approximately 26% of all child recipients. In addition, there are recommendations for the dosing of maintenance immunosuppression in solid organ transplantation for lung transplantation endorsed by the American College of Clinical Pharmacy, the American Society of Transplantation, and the International Society for Heart and Lung Transplantation. However, ethnic differences may lead to differences in drug metabolism, which in turn affects drug administration regimens. There is still no optimal initial dosing schedule, particularly for Chinese children with lung transplantation within normal hematocrit levels. Therefore, the purpose of this study is to optimize the initial tacrolimus dosage in Chinese children with lung transplantation within normal hematocrit levels.

## 2 Methods

### 2.1 Patient and dose design

In a previous study, Cai et al. (23) obtained 807 tacrolimus concentrations from 52 lung transplant patients, and a population pharmacokinetic analysis was performed using a non-linear mixed-effects modeling software (NONMEM; ICON Development Solutions, Ellicott City, MD, USA). The range of age was 16–78, whose median was 54 years old, total body weight was 32–75 kg, hematocrit level was 18–41.7%, alanine transaminase was 2–289 UI^−1^, and aspartate aminotransferase was 5–348 UI^−1^ (23). Patients received an immunosuppressive regimen comprising tacrolimus, mycophenolate mofetil, and corticosteroids (23). They found that body weight, hematocrit, daily dose of tacrolimus, postoperative days, and co-therapy with voriconazole affected tacrolimus clearance (23).

The present study uses a published population pharmacokinetic model of tacrolimus in lung transplantation recipients (23) to extrapolate the dose optimization scheme, which were shown in Equations 1 and 2, where the standard of hematocrit level is fixed at 40% (23, 24).

(1) $$\begin{array}{rl} \mathrm{CL}/F & =13.1\times (\mathrm{WT}/70{)}^{0.75}\times (\mathrm{HCT}/30{)}^{-0.868}\\ & \times (\mathrm{DD}/3{)}^{0.616}\times (\mathrm{POD}/30{)}^{0.0807}\\ & \times 1.3\;(\mathrm{if}\;CYP3A5*1\;\mathrm{carriers})\\ & \times 0.638\;(\mathrm{if}\;\mathrm{with}\;\mathrm{voriconazole}\;\mathrm{comedication}) \end{array}$$

(2) $$V_{d}/F=636\times (\mathrm{WT}/70)$$

WT was body weight, HCT was hematocrit, DD was the daily dose of tacrolimus, POD was postoperative days, and voriconazole was co-therapy with voriconazole (23).

**Figure 1:**
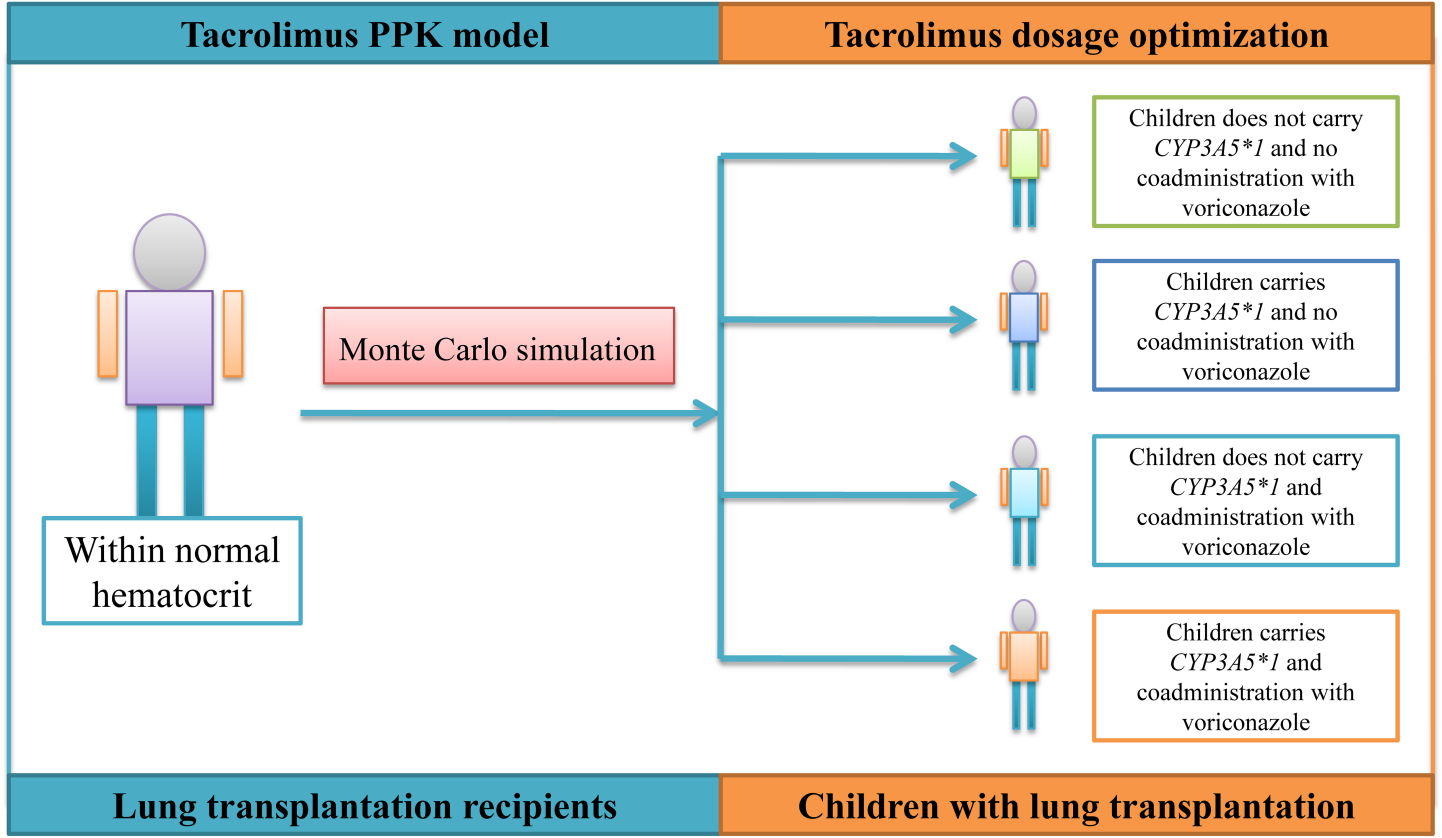
Monte Carlo simulation for four categories.

The simulated patients are divided into four categories: (A) children with lung transplantation who do not carry the CYP3A5*1 gene and have no coadministration with voriconazole; (B) children with lung transplantation who carry the CYP3A5*1 gene and have no coadministration with voriconazole; (C) children with lung transplantation who do not carry the CYP3A5*1 gene and have coadministration with voriconazole; and (D) children with lung transplantation who carry the CYP3A5*1 gene and have coadministration with voriconazole, which are shown in Figure 1. For each category of patients, four different weight groups (10, 20, 30, 40 kg) are simulated, and each weight group is recombined with 14 dosage regimens (0.01, 0.02, 0.03, 0.04, 0.05, 0.06, 0.07, 0.08, 0.09, 0.10, 0.11, 0.12, 0.13, 0.14 mg/kg/day split into two doses). The DD value is not fixed; it is calculated from different dosage regimens combined with the patient's body weight. For example, the DD value is 3 mg for a 30 kg patient simulating a 0.10 mg/kg/day dosage regimen. Based on Cai et al.'s report (23), the time it takes to achieve a steady-state concentration of tacrolimus is on the 8th day after the transplant, as measured by the POD value.

### 2.2 Simulating and optimization

The Monte Carlo simulation, also known as random sampling or statistical testing method, is a branch of computational mathematics that was developed in the mid-1940s to accommodate the development of atomic energy at the time. Due to the limitations of the traditional empirical method in approximating the actual physical process, obtaining satisfactory results is difficult, while the Monte Carlo method can effectively simulate the real physical process, resulting in a solution that closely aligns with reality and yields highly satisfactory results. It is also a computational method based on the methods of probability and statistical theory, and it involves employing random numbers (or more commonly, pseudorandom numbers) to solve various computational problems. The approximate solution of the problem is obtained by associating the problem with a certain probability model and implementing a statistical simulation or sampling with an electronic computer. At present, this method has been greatly developed and applied in the research of individualized drug delivery.

The Monte Carlo simulation is used to simulate tacrolimus dosage regimens 1,000 times at different body weights, in which tacrolimus concentrations reach the therapeutic window of lung transplantation (5–15 ng/ml) as an indicator of efficacy evaluation (25). In addition, the simulation evaluates the probability of exceeding the upper limit of the therapeutic window (15 ng/ml) at the 1,000 simulated concentrations as an evaluation of safety.

## 3 Results

### 3.1 Tacrolimus concentrations

**Figure 2:**
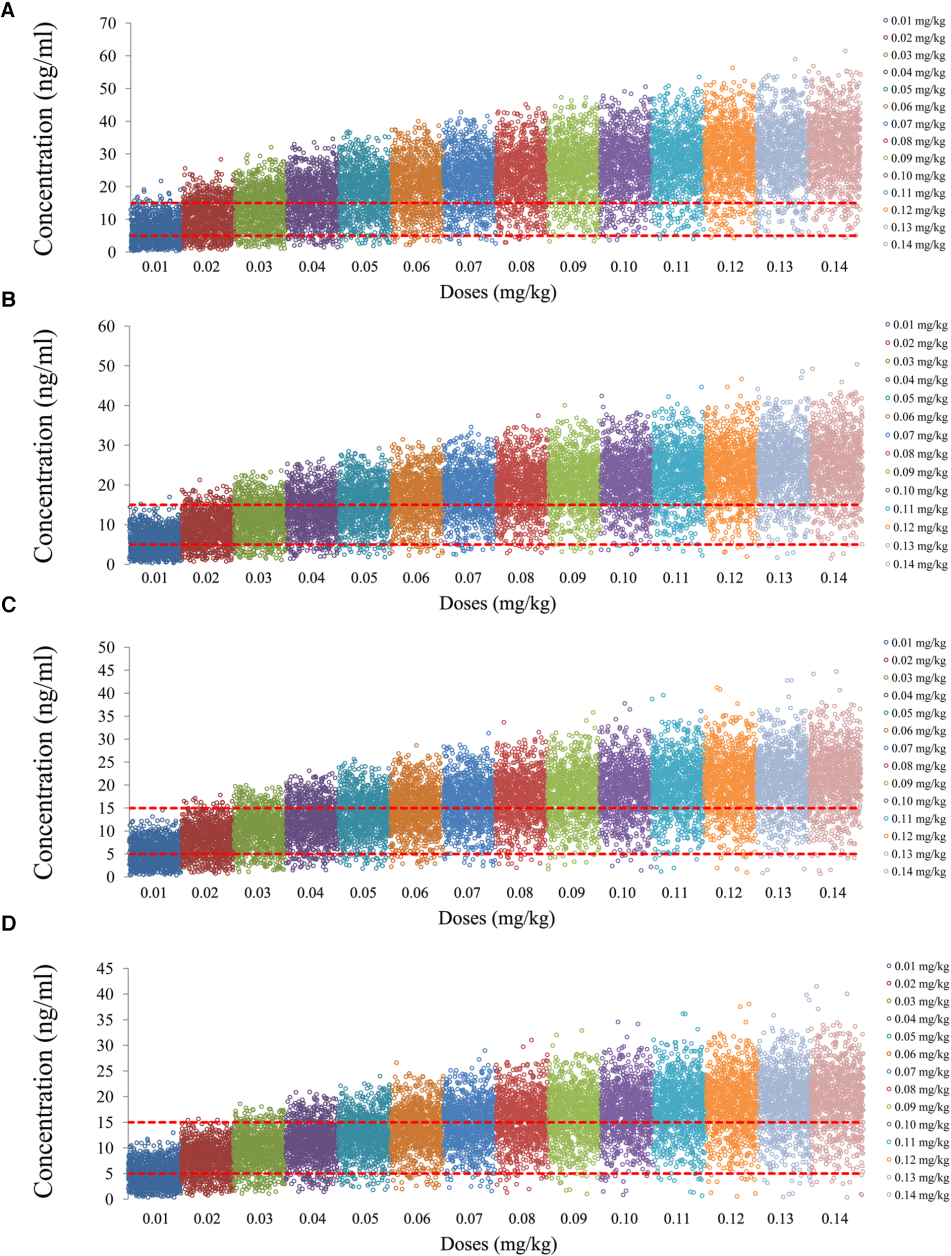
Tacrolimus concentrations of children with lung transplantation who do not carry the CYP3A5*1 gene and have no coadministration with voriconazole. (**A**) Children weighing 10 kg. (**B**) Children weighing 20 kg. (**C**) Children weighing 30 kg. (**D**) Children weighing 40 kg.

**Figure 3:**
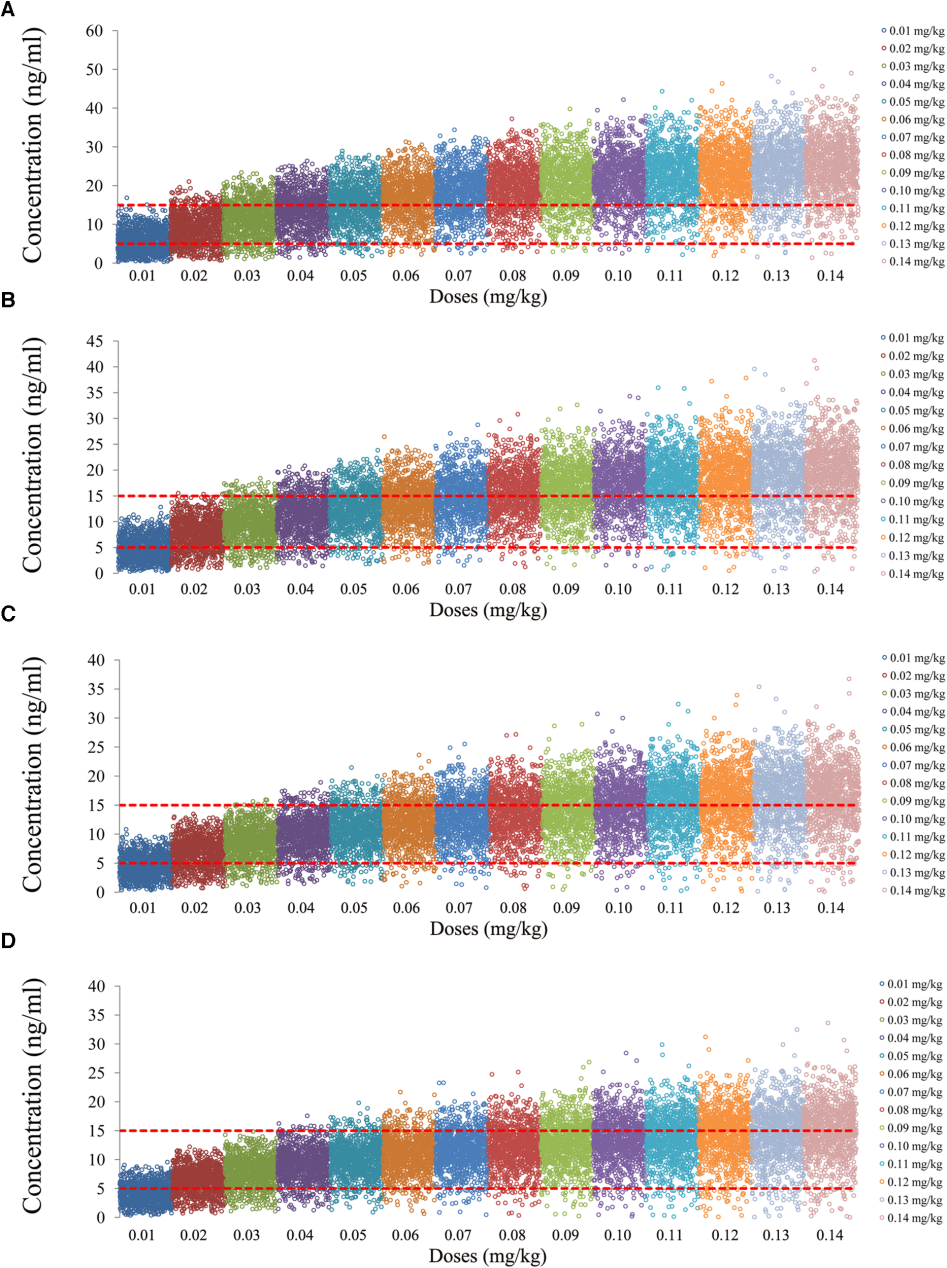
Tacrolimus concentrations of children with lung transplantation who carry the CYP3A5*1 gene and have no coadministration with voriconazole. (**A**) Children weighing 10 kg. (**B**) Children weighing 20 kg. (**C**) Children weighing 30 kg. (**D**) Children weighing 40 kg.

**Figure 4:**
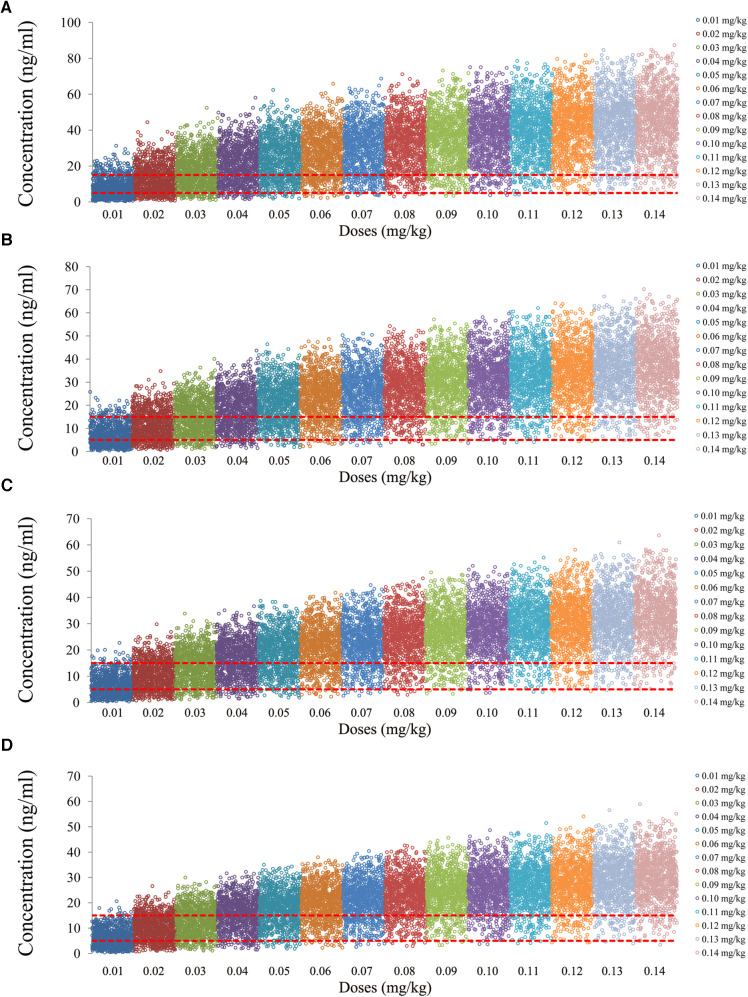
Tacrolimus concentrations of children with lung transplantation who do not carry the CYP3A5*1 gene and have coadministration with voriconazole. (**A**) Children weighing 10 kg. (**B**) Children weighing 20 kg. (**C**) Children weighing 30 kg. (**D**) Children weighing 40 kg.

**Figure 5:**
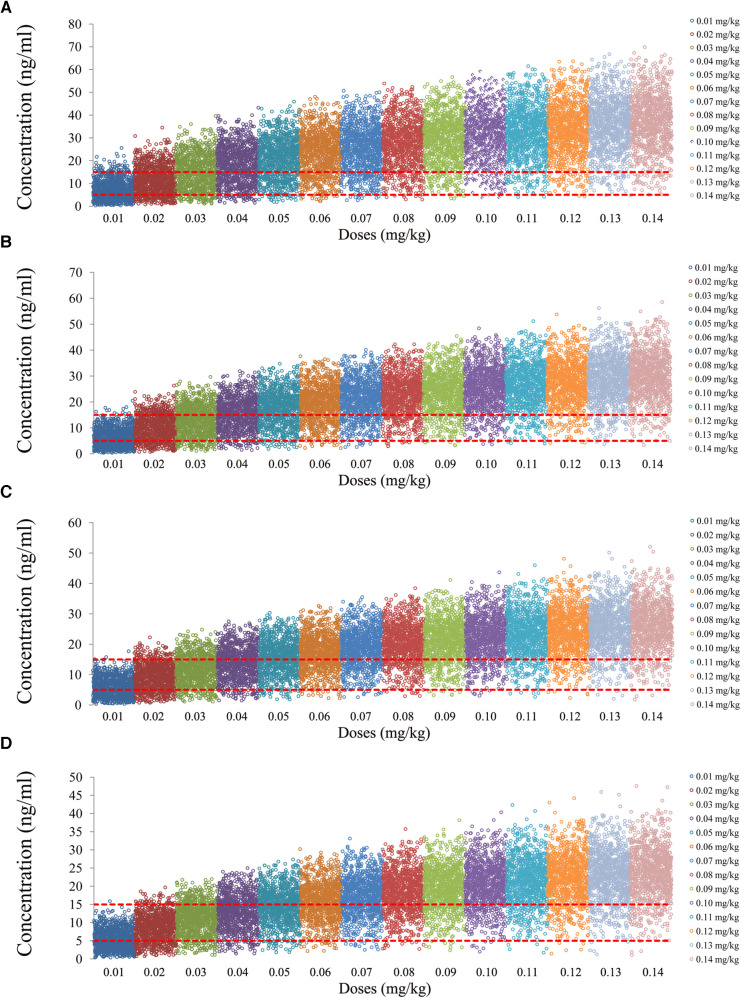
Tacrolimus concentrations of children with lung transplantation who carry the CYP3A5*1 gene and have coadministration with voriconazole. (**A**) Children weighing 10 kg. (**B**) Children weighing 20 kg. (**C**) Children weighing 30 kg. (**D**) Children weighing 40 kg.

The tacrolimus concentrations from the four categories, namely, children with lung transplantation who do not carry the CYP3A5*1 gene and have no coadministration with voriconazole, children with lung transplantation who carry the CYP3A5*1 gene and have no coadministration with voriconazole, children with lung transplantation who do not carry the CYP3A5*1 gene and have coadministration with voriconazole, and children with lung transplantation who carry the CYP3A5*1 gene and have coadministration with voriconazole, are shown in Figures 2–5, respectively. The two red dotted lines are 5 and 15 ng/ml concentration values, respectively. The points located between the two red dotted lines meet the therapeutic window requirement. The points in different colors represent simulated drug concentrations for different dosage regimens, and the vertical coordinate of each point corresponds to the respective drug concentration. According to the color, the simulated dosages from left to right are 0.01, 0.02, 0.03, 0.04, 0.05, 0.06, 0.07, 0.08, 0.09, 0.10, 0.11, 0.12, 0.13, and 0.14 mg/kg/day.

### 3.2 Optimizing the initial tacrolimus dosage

**Figure 6:**
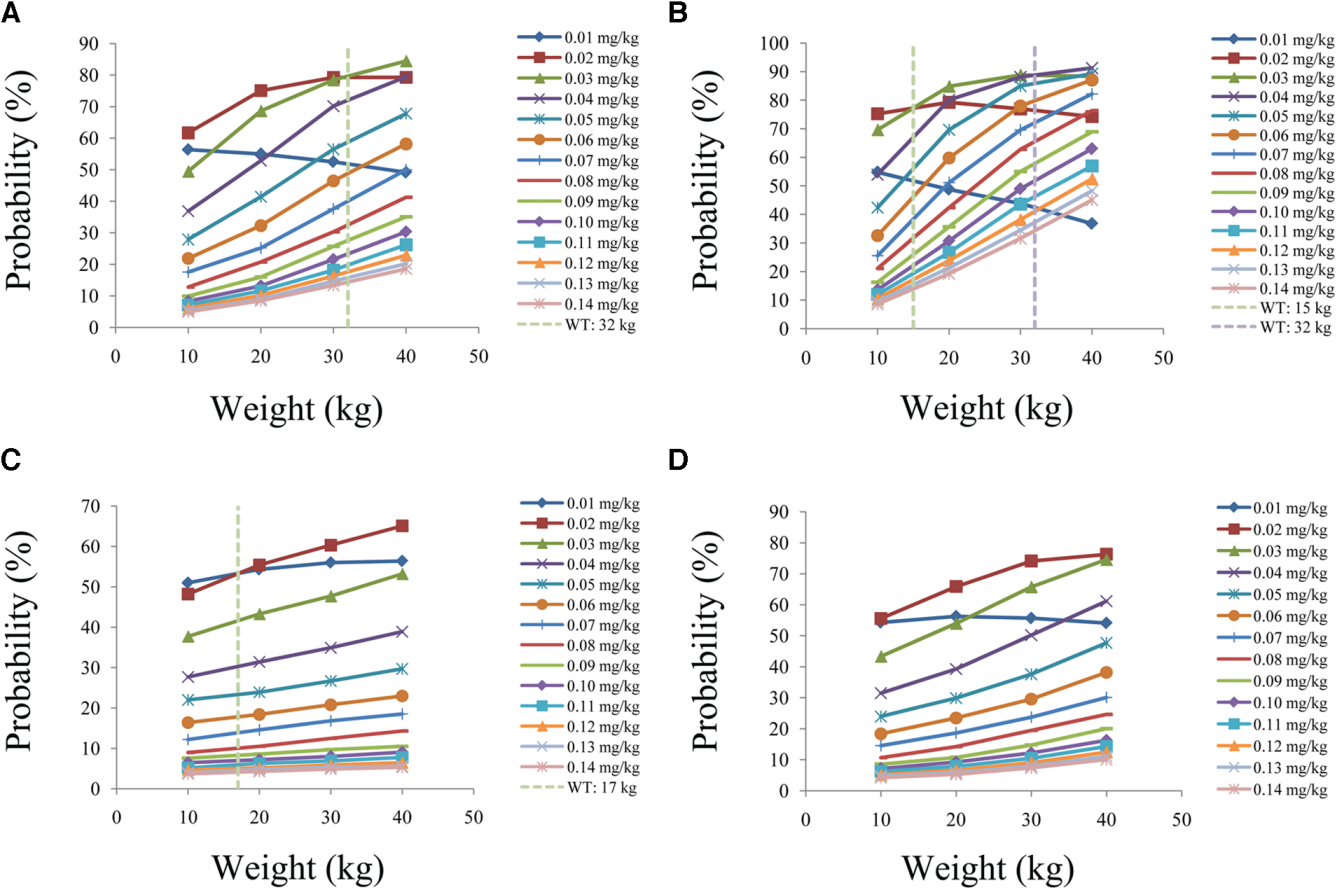
Probability to achieve the target concentrations. (**A**) children with lung transplantation who do not carry the CYP3A5*1 gene and have no coadministration with voriconazole, (**B**) children with lung transplantation who carry the CYP3A5*1 gene and have no coadministration with voriconazole, (**C**) children with lung transplantation who do not carry the CYP3A5*1 gene and have coadministration with voriconazole, and (**D**) children with lung transplantation who carry the CYP3A5*1 gene and have coadministration with voriconazole.

Figure 6 is the probability of tacrolimus concentration reaching the therapeutic window, among which children with lung transplantation who do not carry the CYP3A5*1 gene and have no coadministration with voriconazole, children with lung transplantation who carry the CYP3A5*1 gene and have no coadministration with voriconazole, children with lung transplantation who do not carry the CYP3A5*1 gene and have coadministration with voriconazole, and children with lung transplantation who carry the CYP3A5*1 gene and have coadministration with voriconazole are shown in Figures 6A–D, respectively. For children with lung transplantation who do not carry the CYP3A5*1 gene and have no coadministration with voriconazole, the recommended dosages of tacrolimus are 0.02 and 0.03 mg/kg/day split into two dosages for children weighing 10–32 and 32–40 kg, respectively. For children with lung transplantation who carry the CYP3A5*1 gene and have no coadministration with voriconazole, the recommended dosages of tacrolimus are 0.02, 0.03, and 0.04 mg/kg/day split into two dosages for children weighing 10–15, 15–32, and 32–40 kg, respectively. For children with lung transplantation who do not carry the CYP3A5*1 gene and have coadministration with voriconazole, the recommended dosages of tacrolimus are 0.01 and 0.02 mg/kg/day split into two dosages for children weighing 10–17 and 17–40 kg, respectively. For children with lung transplantation who carry the CYP3A5*1 gene and have coadministration with voriconazole, the recommended dosage of tacrolimus is 0.02 mg/kg/day split into two dosages for children weighing 10–40 kg. Table 1 shows the recommended therapeutic doses.

**Table 1:** Initial dosage recommendation of tacrolimus.

Without voriconazole				With voriconazole			
Not CYP3A5*1 carriers		CYP3A5*1 carriers		Not CYP3A5*1 carriers		CYP3A5*1 carriers	
Body weight (kg)	Dose (mg/kg/day)	Body weight (kg)	Dose (mg/kg/day)	Body weight (kg)	Dose (mg/kg/day)	Body weight (kg)	Dose (mg/kg/day)
10–32	0.02	10–15	0.02	10–17	0.01	10–40	0.02
32–40	0.03	15–32	0.03	17–40	0.02		
		32–40	0.04				

### 3.3 Safety evaluation

**Figure 7:**
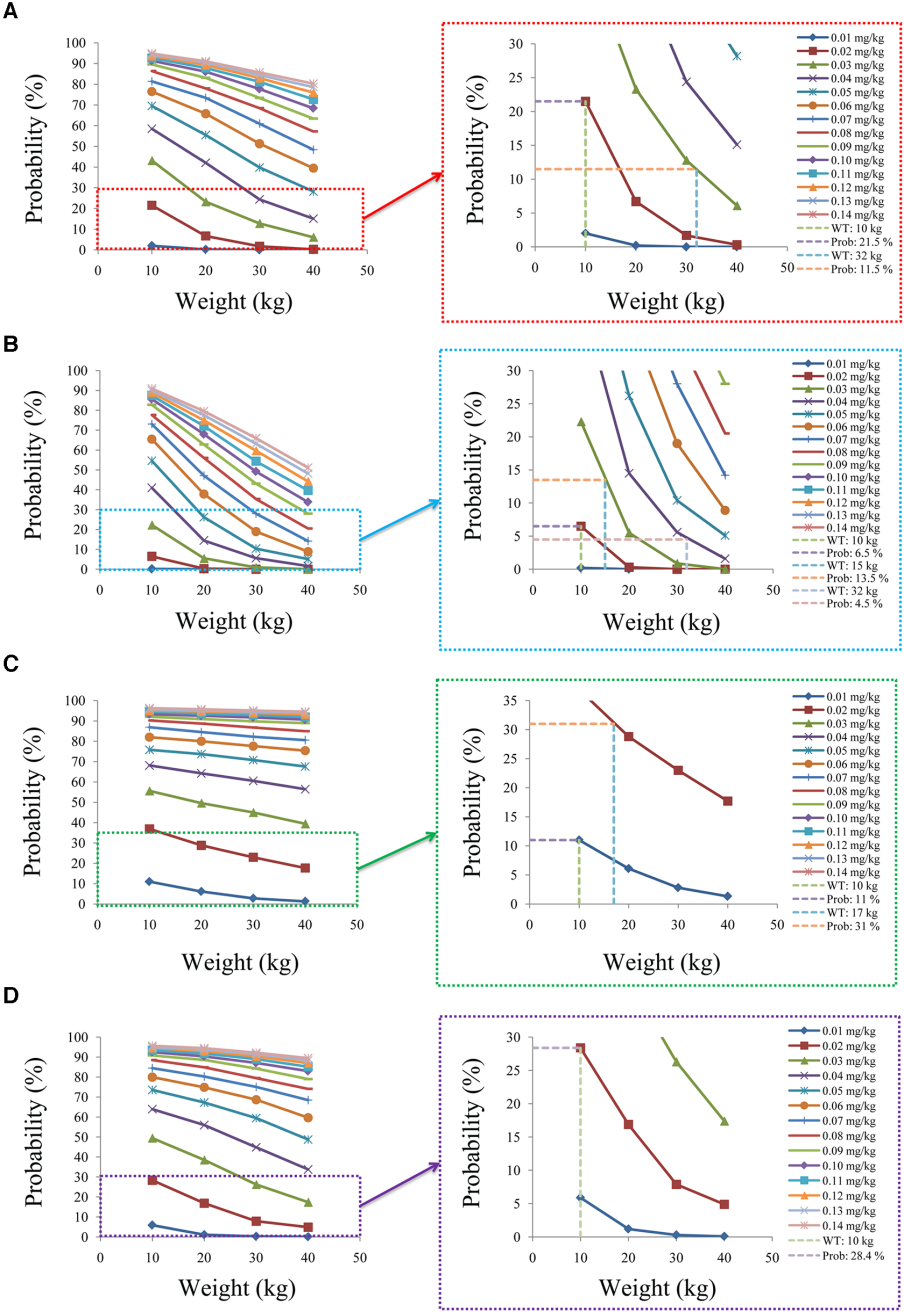
Probability to exceed the upper limit of the target concentrations. (**A**) Children with lung transplantation who do not carry the CYP3A5*1 gene and have no coadministration with voriconazole, (**B**) children with lung transplantation who carry the CYP3A5*1 gene and have no coadministration with voriconazole, (**C**) children with lung transplantation who do not carry the CYP3A5*1 gene and have coadministration with voriconazole, and (**D**) children with lung transplantation who carry the CYP3A5*1 gene and have coadministration with voriconazole.

Figure 7 shows the probability of exceeding the upper limit of the therapeutic window, 15 ng/ml, among which children with lung transplantation who do not carry the CYP3A5*1 gene and have no coadministration with voriconazole, children with lung transplantation who carry the CYP3A5*1 gene and have no coadministration with voriconazole, children with lung transplantation who do not carry the CYP3A5*1 gene and have coadministration with voriconazole, and children with lung transplantation who carry the CYP3A5*1 gene and have coadministration with voriconazole are shown in Figures 7A–D, respectively. For children with lung transplantation who do not carry the CYP3A5*1 gene and have no coadministration with voriconazole, the probabilities of exceeding the upper limit of the target concentrations were less than 21.5% and 11.5%, respectively, for the tacrolimus dosages of 0.02 and 0.03 mg/kg/day, split into two dosages. For children with lung transplantation who carry the CYP3A5*1 gene and have no coadministration with voriconazole, the probabilities of exceeding the upper limit of the target concentrations were less than 6.5%, 13.5%, and 4.5%, respectively, for the tacrolimus dosages of 0.02, 0.03, and 0.04 mg/kg/day, split into two dosages. For children with lung transplantation who do not carry the CYP3A5*1 gene and have coadministration with voriconazole, the probabilities of exceeding the upper limit of the target concentrations were less than 11% and 31%, respectively, for the tacrolimus dosages of 0.01 and 0.02 mg/kg/day, split into two dosages. For children with lung transplantation who carry the CYP3A5*1 gene and have coadministration with voriconazole, the probabilities of exceeding the upper limit of the target concentrations were less than 28.4% for the tacrolimus dosage of 0.02 mg/kg/day, split into two dosages.

## 4 Discussion

FK-506-binding protein 12 (FKBP-12) is an important immunophilin targeted by tacrolimus in T cells, and tacrolimus can form a complex with FKBP-12, thus repressing the phosphatase calcineurin, an enzyme necessary to activate the nuclear factor of T cells (NF-AT) (26, 27). Currently, the use of tacrolimus in combination with mycophenolate mofetil and corticosteroids can maintain an immunosuppressive regimen in approximately 60% of patients, and it is preferred over cyclosporine A, owing to its higher long-term graft and patient survival rates (23, 28).

Tacrolimus is a commonly used immunosuppressant in clinical practice that can cure a variety of diseases, such as nephrotic syndrome (29), myasthenia gravis (30), ulcerative colitis (31), systemic lupus erythematosus (32), lupus nephritis (33), and autoimmune hepatitis (34). In addition, tacrolimus also plays an important role in preventing organ rejection after transplantation. For example, tacrolimus is widely used in hematopoietic stem cell transplantation (8), liver transplantation (7), kidney transplantation (6), heart transplantation (4), and lung transplantation (5). Due to the widespread use and lifelong medication requirement of tacrolimus, its adverse effects, which primarily include nephrotoxicity (27), neurotoxicity, infection, and post-transplantation diabetes mellitus (35), have become a clinical problem that cannot be ignored.

Tacrolimus, despite its critical function in preventing rejection during organ transplants (4–7), presents a challenge in terms of personalized administration for clinical use. This is also one of the more difficult clinical issues, as lower concentrations reduce the immunosuppressive effect of tacrolimus, while higher concentrations tend to increase the risk of tacrolimus-related toxicity (9, 10). At present, the clinical approach to solving this problem is to adjust the next dose of tacrolimus by monitoring drug concentration so that the tacrolimus concentration can reach the therapeutic window; however, this traditional method fails to address the issue of the initial dose. The encouraging thing is that population pharmacokinetics and Monte Carlo simulation offer effective solutions to this difficult problem, which have been demonstrated by many clinical examples, such as initial dose optimization of tacrolimus for children with systemic lupus erythematosus (17), initial dosage optimization of tacrolimus in Chinese pediatric patients undergoing kidney transplantation (20), optimization of the initial dosing scheme of tacrolimus in pediatric refractory nephrotic syndrome patients (36), and population pharmacokinetics and pharmacogenomics of tacrolimus in Chinese children receiving a liver transplant: initial dose recommendation (37). However, the initial dosage of tacrolimus in Chinese children with lung transplantation within normal hematocrit levels remains unknown. The purpose of this study is to optimize the initial tacrolimus dosage in Chinese children with lung transplantation within normal hematocrit levels.

Cai et al.'s (23) model is developed in patients older than 16 years, where body weight is allometrically included in both tacrolimus clearance and distribution volume. The current simulations are considering children with lung transplantation, which is the scientific category of pediatric extrapolation. In the present study, for children with lung transplantation who do not carry the CYP3A5*1 gene and have no coadministration with voriconazole, the recommended dosages of tacrolimus are 0.02 and 0.03 mg/kg/day, split into two dosages, for children weighing 10–32 and 32–40 kg, respectively. For children with lung transplantation who carry the CYP3A5*1 gene and have no coadministration with voriconazole, the recommended dosages of tacrolimus are 0.02, 0.03, and 0.04 mg/kg/day, split into two dosages, for children weighing 10–15, 15–32, and 32–40 kg, respectively. For children with lung transplantation who do not carry the CYP3A5*1 gene and have coadministration with voriconazole, the recommended dosages of tacrolimus are 0.01 and 0.02 mg/kg/day, split into two dosages, for children weighing 10–17 and 17–40 kg, respectively. For children with lung transplantation who carry the CYP3A5*1 gene and have coadministration with voriconazole, the recommended dosage of tacrolimus is 0.02 mg/kg/day, split into two dosages, for children weighing 10–40 kg, respectively.

In addition, this study also further analyzed the safety aspect. For children with lung transplantation who do not carry the CYP3A5*1 gene and have no coadministration with voriconazole, the probabilities of exceeding the upper limit of the target concentrations were less than 21.5% and 11.5%, respectively, for the tacrolimus dosages of 0.02 and 0.03 mg/kg/day, split into two dosages. For children with lung transplantation who carry the CYP3A5*1 gene and have no coadministration with voriconazole, the probabilities of exceeding the upper limit of the target concentrations were less than 6.5%, 13.5%, and 4.5%, respectively, for the tacrolimus dosages of 0.02, 0.03, and 0.04 mg/kg/day, split into two dosages. For children with lung transplantation who do not carry the CYP3A5*1 gene and have coadministration with voriconazole, the probabilities of exceeding the upper limit of the target concentrations were less than 11% and 31%, respectively, for the tacrolimus dosages of 0.01 and 0.02 mg/kg/day, split into two dosages. For children with lung transplantation who carry the CYP3A5*1 gene and have coadministration with voriconazole, the probabilities of exceeding the upper limit of the target concentrations were less than 28.4% for the tacrolimus dosage of 0.02 mg/kg/day, split into two dosages.

Physiologically, age and body mass index (BMI) may have a certain impact on the clearance of tacrolimus. In addition, weight, age, and BMI are related to a certain extent, so in the present study, we can indirectly reflect the influence of age and BMI on tacrolimus through the analysis of body weight. Importantly, the pediatric tacrolimus population pharmacokinetic model has included more studies that have analyzed body weight as a covariable (17, 36). In addition, Liu et al. (38) found that a composite *CYP3A* phenotype incorporating both the increase and decrease variant information from *CYP3A4* in addition to *CYP3A5* may significantly influence tacrolimus C_0_/D during the early postoperative period. In the study conducted by Cai et al. (20), *CYP3A4* was not analyzed as a factor in the population pharmacokinetic model of tacrolimus in lung transplantation; however, in pediatric patients undergoing kidney transplantation and pediatric patients undergoing liver transplantation (37), the analysis of *CYP3A4* was conducted, but *CYP3A4* was not included in the final tacrolimus model.

In addition, we explored in a previous study the optimization of the initial dose regimen of tacrolimus in pediatric lung transplant recipients within a lower hematocrit level of 30% (39). “Without voriconazole, the tacrolimus doses recommended for paediatric lung transplant recipients who were not CYP3A5*1 carriers were 0.02, 0.03, and 0.04 mg/kg/day, split into two doses, for weights of 10–16, 16–30, and 30–40 kg, respectively. For paediatric lung transplant recipients who were CYP3A5*1 carriers, the tacrolimus doses of 0.03, 0.04, 0.05, and 0.06 mg/kg/day, split into two doses, were recommended for weights of 10–16, 16–25, 25–30, and 30–40 kg, respectively. With voriconazole, the tacrolimus dose recommended for paediatric lung transplant recipients who were not CYP3A5*1 carriers was 0.02 mg/kg/day, split into two doses, for weights of 10–40 kg. For paediatric lung transplant recipients who were CYP3A5*1 carriers, tacrolimus doses of 0.02 and 0.03 mg/kg/day, split and two doses, were recommended for weights of 10–24 and 24–40 kg, respectively” (39). In comparison to the previous study, the present study found that changes in the hematocrit levels affected the initial dose recommendation of tacrolimus in pediatric lung transplant patients. Thus, hematocrit should be considered in the initial administration of tacrolimus to pediatric lung transplant recipients.

The current extrapolation-based model used in this study was derived from adult lung transplant patients in China, so caution should be exercised when applying the recommended pediatric lung transplant dose to other populations (Caucasian, Black), due to the potential variation in drug clearance among different races. Since we are interested in a state after lung transplantation, which mainly refers to antirejection therapy by tacrolimus after lung transplantation, all indications for transplantation can be considered, similar to the published model (23) we referred to.

Currently, there is limited research on the optimal dosage recommendation for tacrolimus in Chinese pediatric lung transplant patients; few studies have been reported. It is the first time for the present study to optimize the initial tacrolimus dosage in children with lung transplantation within normal hematocrit. Of course, the optimized dosage recommendation should be compared with future research to verify the reliability of our conclusions.

## 5 Conclusion

It is the first time to optimize the initial tacrolimus dosage in Chinese children undergoing lung transplantation with normal hematocrit levels, and further pediatric clinical studies are needed to validate the findings of this study.

## Data Availability

The original contributions presented in the study are included in the article/Supplementary Material, further inquiries can be directed to the corresponding author.
